# Apoptosis-Specific Protein (ASP) Identified in Apoptotic *Xenopus* Thymus Tumor Cells

**DOI:** 10.1155/1998/70616

**Published:** 1998

**Authors:** John Horton, Anne Milner, Trudy Horton, Pamela Ritchie, Duncan Gascoyne, Tim Hewson, Ester Hammond, Christopher Gregory, Roger Grand

**Affiliations:** 1 Department of Biological Sciences University of Durham Durham DH1 3LE United Kingdom; 2 CRC Institute for Cancer Studies University of Birmingham Medical School Birmingham B15 2TJ United Kingdom; 3 Department of Biochemistry Queen's Medical Centre University of Nottingham NG7 2UH USA

**Keywords:** Apoptosis-specific protein (ASP) evolution, apoptosis detection by flow cytometry, lymphoid tumor cells, Xenopus immune system

## Abstract

A novel apoptosis-specific protein (ASP) has recently been identified in the cytoplasm of apoptotic mammalian cells. This paper investigates whether ASP is found in *Xenopus* thymus tumor-derived lymphoid cell lines undergoing apoptosis and also in apoptotic, nontransformed splenocytes. Cultured *Xenopus* tumor lymphoid cells induced to undergo, apoptosis by serum deprivation or treatment with the calcium ionophore, ionomycin, displayed altered morphology typical of apoptotic cells, as judged by flow cytometric light-scatter characteristics and by fluorescence microscopy of acridine-orange-stained cells. Flow cytometry of permeabilized cells and fluorescence microscopy of acetone-fixed cytospins revealed that apoptotic *Xenopus* tumor cells, especially those displaying loss or condensation of DNA, displayed increased expression of epitopes recognized by a rabbit polyclonal antibody against ASP. Flow cytometry confirmed that ASP is also expressed in splenocytes induced to apoptose by culture in ionomycin or following concanavalin A stimulation. No increased expression of ASP was seen when lymphoid tumor cells or splenocytes were induced into necrosis by overdose with the antifungal agent amphotericin B. Western blotting with antibody against ASP identified the emergence of several protein bands in cell lysates from apoptotic, but not necrotic, *Xenopus* tumor cells. The new and simple methodology for identifying apoptotic cells described here is likely to be of value to those studying immune system development and associated programmed cell death in *Xenopus*.


*Developmental Immunology*, 1998, Vol. 5, pp. 333–348
Reprints available directly from the publisher
Photocopying permitted by license only

© 1998 OPA (Overseas Publishers Association)
N.V. Published by license under the
Harwood Academic Publishers imprint,
part of the Gordon and Breach Publishing Group
Printed in Malaysia

### Apoptosis-Specific Protein (ASP) Identified in Apoptotic *Xenopus* Thymus Tumor Cells

JOHN HORTON[a,*], ANNE MILNER[b], TRUDY HORTON[a], PAMELA RITCHIE[a], DUNCAN GASCOYNE[a], TIM HEWSON[a], ESTER HAMMOND[b], CHRISTOPHER GREGORY[c] and ROGER GRAND[b]

[a]*Department of Biological Sciences, University of Durham, Durham DH1 3LE, UK;* [b]*CRC Institute for Cancer Studies, University of Birmingham Medical School, Birmingham B15 2TJ, UK;* [c]*Department of Biochemistry, Queen's Medical Centre, University of Nottingham NG7 2UH, UK*

(*Received 24 August 1997; In final form 5 January 1998*)

A novel apoptosis-specific protein (ASP) has recently been identified in the cytoplasm of apoptotic mammalian cells. This paper investigates whether ASP is found in *Xenopus* thymus tumor-derived lymphoid cell lines undergoing apoptosis and also in apoptotic, nontransformed splenocytes. Cultured *Xenopus* tumor lymphoid cells induced to undergo apoptosis by serum deprivation or treatment with the calcium ionophore, ionomycin, displayed altered morphology typical of apoptotic cells, as judged by flow cytometric light-scatter characteristics and by fluorescence microscopy of acridine-orange-stained cells. Flow cytometry of permeabilized cells and fluorescence microscopy of acetone-fixed cytospins revealed that apoptotic *Xenopus* tumor cells, especially those displaying loss or condensation of DNA, displayed increased expression of epitopes recognized by a rabbit polyclonal antibody against ASP. Flow cytometry confirmed that ASP is also expressed in splenocytes induced to apoptose by culture in ionomycin or following concanavalin A stimulation. No increased expression of ASP was seen when lymphoid tumor cells or splenocytes were induced into necrosis by overdose with the antifungal agent amphotericin B. Western blotting with antibody against ASP identified the emergence of several protein bands in cell lysates from apoptotic, but not necrotic, *Xenopus* tumor cells. The new and simple methodology for identifying apoptotic cells described here is likely to be of value to those studying immune system development and associated programmed cell death in *Xenopus*.

*Keywords:* Apoptosis-specific protein (ASP) evolution, apoptosis detection by flow cytometry, lymphoid tumor cells, *Xenopus* immune system

### INTRODUCTION

Apoptosis plays a crucial role in the development, physiology, and pathology of the immune system (Gregory, 1995). During ontogeny, T and B lymphocytes that are autoreactive or nonfunctional, due to inappropriate rearrangement of antigen-receptor gene segments, die by apoptosis (Osborne, 1996). Cytolytic lymphocytes (both T and NK cells) kill by inducing apoptosis in their target cells (Berke, 1995), a

*Corresponding author. Tel.: +44 191 374 3359. Fax: +44 191 374 2417. E-mail: J.D. Horton@durham.ac.uk mechanism that, unlike necrosis, prevents inflammation or spilling of cellular contents that might damage or infect surrounding healthy cells. Inappropriate apoptosis of lymphocytes may be triggered in some pathologies, for example, AIDS (Duke, 1996).

In mammals, apoptosis can be characterized morphologically by cell shrinkage, nuclear pycnosis, chromatin condensation, and plasma membrane blebbing, with rapid phagocytosis of apoptotic cells (Martins and Earnshaw, 1997). A cascade of molecular and biochemical events is also associated with apoptosis (An and Knox, 1996; Nagata, 1997), including the interaction between apoptosis-signaling cell-surface TNF-receptor family members (such as *fas*) and their ligands (e.g., *fas* ligand), subsequent intracellular signaling by death factors such as the caspase family of cysteine proteases, and the fragmentation and loss of DNA. Recently, a novel "apoptosis-specific protein" (ASP) has been identified in human Burkitt lymphoma cells and in adenovirus-transformed human and rat embryo cells (Grand et al., 1995). ASP is expressed when these cells are triggered to apoptose by a variety of stimuli, including serum deprivation and exposure to ionomycin. ASP was identified using a rabbit polyclonal antibody raised against a synthetic peptide of the human *c-jun* protein, which cross reacts with ASP. Western blotting revealed that the principal peptide (ASP) detected by this antibody in apoptotic cells had a molecular weight of 45-kD, rather than the 39-kD bona fide *c-jun* protein. ASP is found in the cytoplasm, whereas *c-jun*, a component of the activator protein 1 (AP-1) transcription factor, is located in the nucleus. Increased ASP expression in apoptotic cells appears to be attributable to an increase in translation of preexisting mRNA (Hammond et al., 1998). ASP persists long after cell death and appears to form part of a modified cytoskeleton perhaps responsible for maintenance of membrane integrity in apoptosing cells prior to their phagocytosis (Grand et al., 1995).

Since evolutionary conservation of the molecular basis of apoptosis is indicated from comparative studies of programmed cell death from mammals back to invertebrates such as nematodes (Martins and Earnshaw, 1997), it might be expected that apoptosis at the amphibian level of evolution can be identified by probes used for mammalian apoptosis. In this paper, we investigate whether the anti-ASP antibody used by Grand et al. (1995) can identify conserved apoptosis-specific proteins in *Xenopus*, whose immune system development is being explored in depth (Du Pasquier et al., 1996; Horton et al., 1996a). Our studies center on *Xenopus* thymus tumor-derived lymphoid-cell lines $B_3B_7$ and 15/0 (Du Pasquier and Robert, 1992; Robert et al., 1994), since novel apoptotic markers for these tumor cells, being used as targets for *Xenopus* cytolytic lymphocytes (for both NK-like cells [Horton et al., 1996b], and T cells [Horton et al, 1997a, 1997b]) would be of particular value. The applicability of our findings to non-transformed *Xenopus* cells is also briefly addressed, by monitoring ASP in apoptotic splenic lymphocytes.

### RESULTS

Experiments on lymphoid tumor cells were carried out at room temperature (18-20°C), and those on splenocytes at 27°C, as explained in the Materials and Methods section.

### Serum Deprivation Induces Apoptosis and ASP Staining

$B_3B_7$ cells were kept for up to 336 hr (14 days) in medium without subculture, in order to investigate the effects of serum deprivation. Samples were periodically removed for flow cytometric and fluorescence microscopic analysis. "Viable" cell concentration assessed by microscopy (trypan blue exclusion) initially increased from 2-3 $\times$ $10^5$/ml at time of seeding to ~1-2 $\times$ $10^6$/ml by 168 hr; by 336 hr, viable cell numbers had diminished as dead cells and cell debris became increasingly evident.

Samples were analyzed by flow cytometry for alterations in cell size (FS) and granularity (SS). Figure 1 shows that at the time of seeding, very few $B_3B_7$ cells were gated as apoptotic, whereas by 336 hr of culture, around 50% cells displayed reduced FS signal but increased SS signal, features that in mammals are indicative of apoptosis (Milner et al., 1995).

Aliquots were also taken for fluorescence microscopic analysis of either acetone-fixed cytospins stained with anti-ASP (Figure 2E) or unfixed cells stained with acridine orange (Figures 2A to 2D). Replicate experiments revealed <5% ASP-expressing ($ASP^+$) cells at the start of culture; by 168 hr, $ASP^+$ cells still represented <10%, but this figure consistently rose to around 40% by 336 hr of culture under serum-deprivation conditions. The ASP antibody stains a cytoplasmic epitope within the $B_3B_7$ cells (Figure 2E). Counterstaining of cytospins with the DNA-binding fluorochrome propidium iodide (PI) revealed that all $ASP^+$ cells possessed either pycnotic nuclei or loss of nuclear material; conversely, all the cells with pycnotic nuclei (i.e., with classical morphologic apoptotic criteria [Kerr et al., 1972]) were $ASP^+$. Moreover, the percentage of $ASP^+$ cells recorded in cytospins was directly comparable to the proportion of unfixed cells scored as apoptotic on the basis of acridine-orange staining (data not shown).

### Comparison of ASP Staining Following Ionomycin-Induced Apoptosis and Amphotericin-Induced Necrosis

$B_3B_7$ cells were either cultured in medium containing 10 $\mu$g/ml ionomycin or 50 $\mu$g/ml amphotericin B.

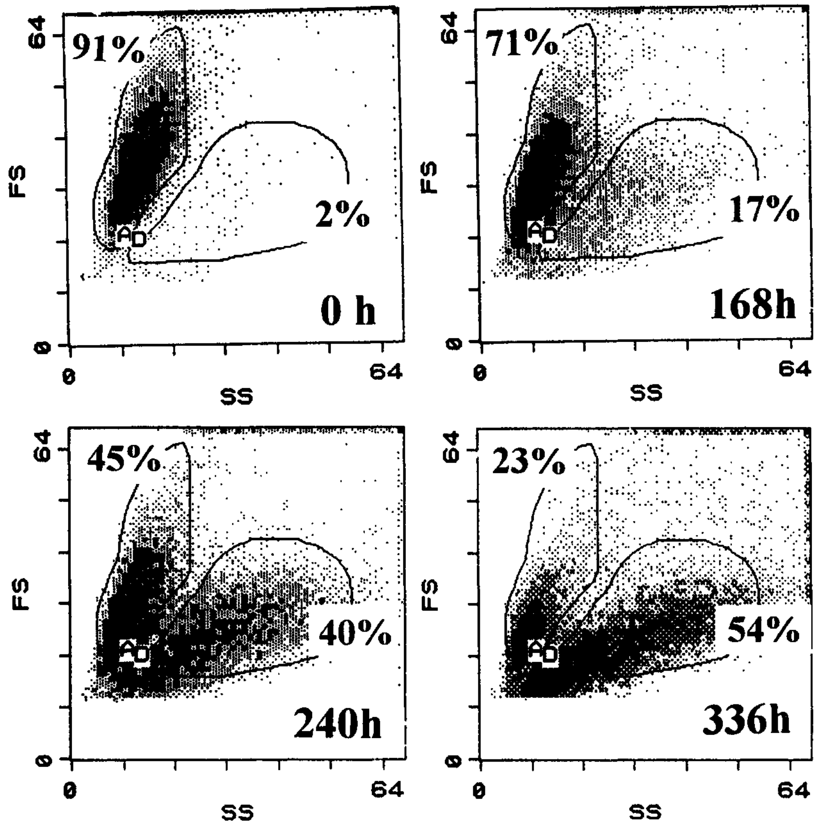

FIGURE 1 Induction of apoptosis through serum deprivation. The series of representative flow cytometer histograms show changes in forward scatter (FS) and side scatter (SS) of $B_3B_7$ *Xenopus* tumor cells (cultured in 10 ml tumor medium with no medium change) sampled at 7 days (168 hr), 10 days, and at 14 days (336 hr). With time after seeding (0 hr), the events recorded shift from region A ("viable" cells) to region D ("apoptotic" cells), as cells come to display reduced FS signal and increased SS signal. Ten thousand events were recorded per histogram.

with saponin to allow cytoplasmic staining. The percentage of dead cells with leaky cell membranes was also estimated by flow cytometric analysis of PI stained, nonsaponin-treated samples (see Figure 3).

### Ionomycin Treatment (Figure 3A)

Fluorescence microscopic analysis of cytospins revealed a sharp increase in ASP-expressing cells between 24 hr (<5%) and 72 hr (approaching 20%) following calcium ionophore treatment, the level of ASP then appearing to plateau between 72 and 168 hr. $ASP^+$ cytospun cells, which were counterstained with PI, consistently displayed pycnotic nuclei typical of apoptosing cells. Flow cytometry of saponin-treated cells revealed higher levels of ASP staining compared to the cytospin data, but showed the same trend in ASP expression. Thus, about 20% $ASP^+$ cells were recorded by cytometry when cultures were seeded and again at 24 hr following ionomycin treatment, whereas the level of $ASP^+$ cells plateaued at around 50% from 72 to 168 hr. Since PI staining of nonsaponin-treated tumor cells indicated a rapid rise in cells with leaky plasma membranes only *after* 72 hr of ionomycin treatment (from <20% to around 50% at 168 hr incubation), it is evident that the high ASP level recorded (with the cytometer) at 72 hr precedes loss of cell-membrane integrity. Lack of correlation between cell-membrane leakiness and ASP staining is also indicated by dual staining of nonpermeabilized tumor cells with PI and with the ASP antibody, where the $PI^+$ population always contained both $ASP^+$ and $ASP^-$ cells (data not shown).

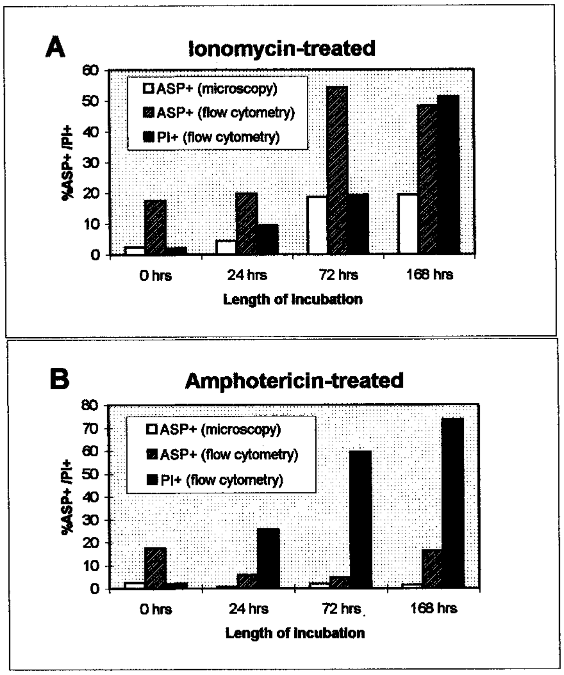

FIGURE 3 Graphs to show proportions of $ASP^+$ and $PI^+$ $B_3B_7$ cells at various times after culture with either (A) ionomycin (10 $\mu$g/ml) or (B) with 50 $\mu$g/ml amphotericin B. The percentage of $ASP^+$ cells was measured by binding with rabbit anti-ASP primary antibody and visualized by FITC-conjugated anti-rabbit Ig antibody, cells having been either transferred to slides and acetone-fixed (for fluorescence microscopy) or unfixed suspensions saponin-treated (for flow cytometry). The percentage of dead cells ($PI^+$) with leaky plasma membranes was also estimated by flow cytometry using nonsaponin-treated, unfixed cells. The fluorescence microscopy data were obtained by counting >500 cells from at least 10 fields of coded cytospins. Flow cytometric data were based on 10,000 events. Data are representative of three separate experiments performed.

### Amphotericin B Treatment (Figure 3B)

When used at a dose 20-fold higher than is effective as a fungicide, amphotericin appears to induce necrosis of tumor cells, since >50% of cells are dead ($PI^+$) within 72 hr of treatment and around 70% display leaky cell membranes by 168 hr. Fluorescence microscopy of cytospins revealed no increase in proportion of ASP-containing cells over the 7-day culture period and few cells with pycnotic nuclei (data not shown). This lack of ASP staining following amphotericin treatment was confirmed by flow cytometry of saponin-treated cells; indeed, a lower percentage of amphotericin-treated cells expressed ASP than at the start of culture. This was probably because no further apoptoses were induced and initial ASP-containing cells were lost.

### ASP Detection by Flow Cytometry

#### *$B_3B_7$ Tumor Cells (Figure 4).*

Flow cytometric traces highlight changes in ASP staining patterns of $B_3B_7$ cells cultured without medium change over a 7-day period. Cells were saponin-treated prior to staining with ASP antibody and the percentages of $ASP^+$ cells shown determined by positive analysis. Such analysis sets markers to exclude 98% cells stained with normal rabbit serum (NRS). Permeabilization tended to result in considerable nonspecific binding of rabbit serum (both anti-

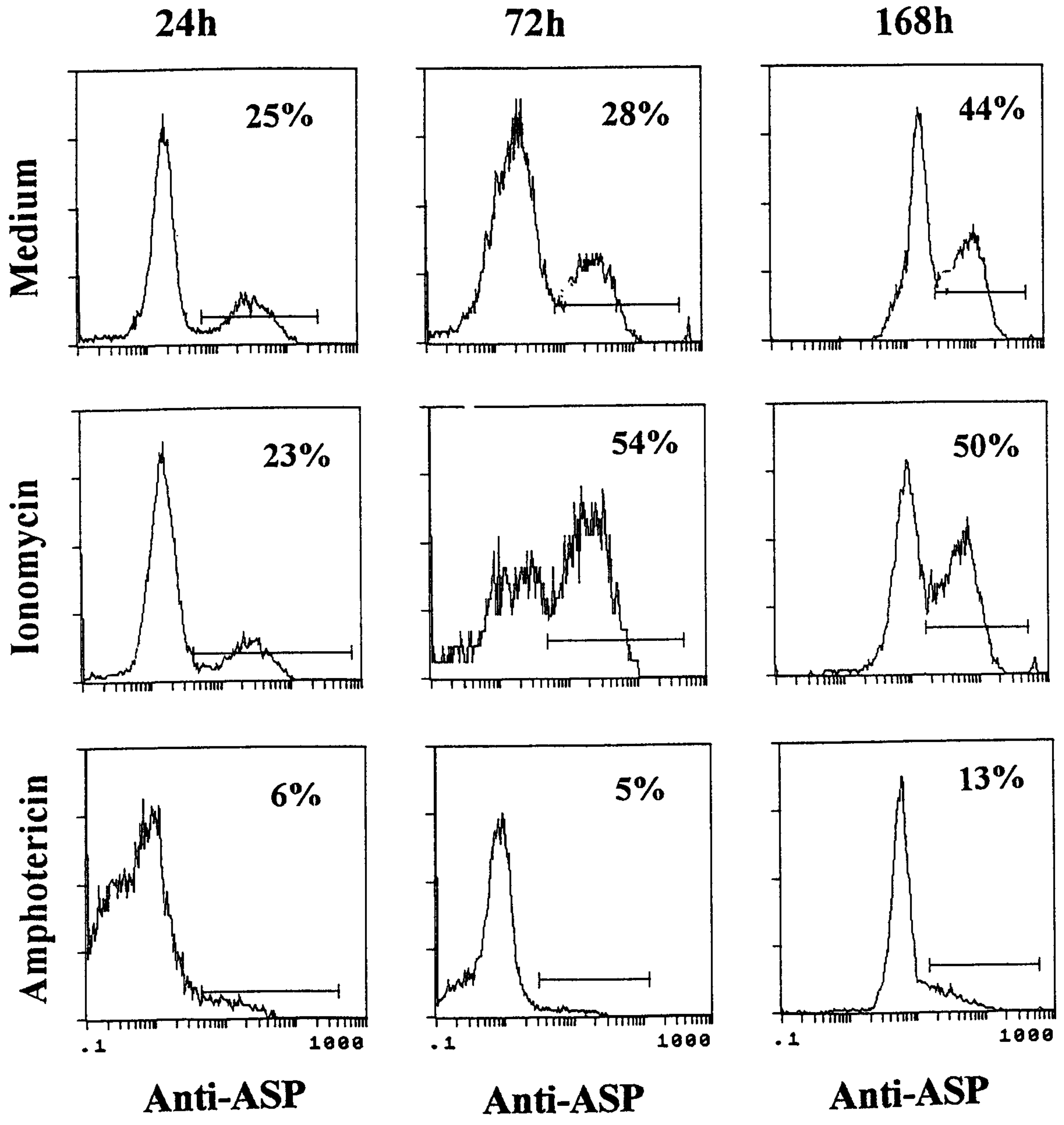

FIGURE 4 Representative flow cytometric traces comparing the percentages of $ASP^+$ $B_3B_7$ cells in samples taken at 24, 72, and 168 hr after seeding to culture flasks. The thymus tumor cells were cultured either in tumor medium alone (serum-deprivation control) or in 10 $\mu$g/ml of ionomycin (to induce apoptosis) or 50 $\mu$g/ml of amphotericin B (to induce necrosis). Cells were permeabilized with saponin prior to staining with rabbit anti-ASP. The markers are set to exclude from positive analyses 98% of the cells stained with irrelevant primary antibody (normal rabbit serum).

ASP and NRS), which caused a shift of both the $ASP^+$ and $ASP^-$ peaks to higher fluorescence intensities (see Figure 4). Cells cultured in medium alone and those treated with 10 $\mu$g/ml ionomycin displayed distinct peaks of ASP-specific fluorescence, ionomycin inducing a more rapid effect than serum deprivation alone. Cells induced to necrose by 50 $\mu$g/ml amphotericin B failed to show elevated ASP staining.

### *Splenocytes (Figure 5 and Figure 6).*

Splenocytes were also assessed by flow cytometry for emergence of ASP during apoptosis. Figure 5 shows the outcome of 72 hr *in vitro* culture of splenocytes in medium alone or in medium containing either ionomycin or amphotericin. Splenocytes cultured in medium alone, then permeabilized with saponin prior to staining with anti-ASP antibody, displayed 20% $ASP^+$ cells, whereas 35% were $ASP^+$ after ionomycin treatment. Amphotericin-induced necrotic cells remained $ASP^-$. Figure 6 reveals that after 7 days culture in medium, 35% splenocytes were now $ASP^+$, whereas 70% of those cultured in Con A had become $ASP^+$. Extensive apoptosis in Con A-treated cultures was also indicated by light-scatter analysis of non-permeabilized cells (data not shown).

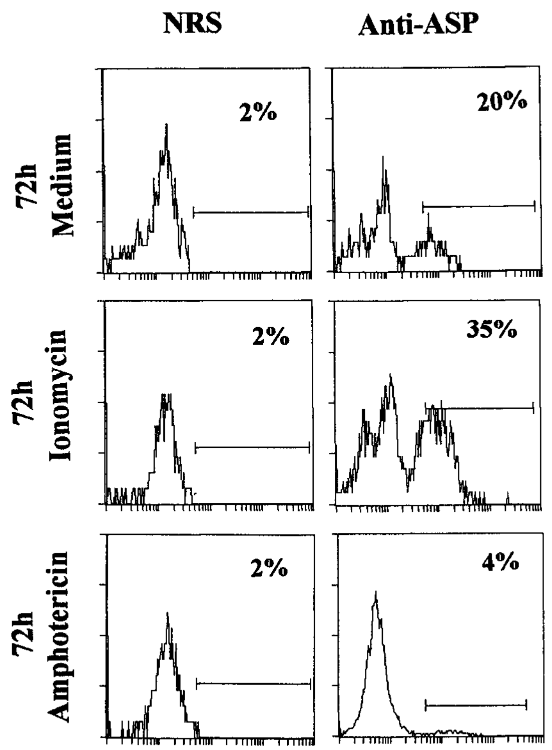

FIGURE 5 Representative flow cytometric traces comparing the percentages of $ASP^+$ splenocytes following culture for 72 hr in either medium, 1 $\mu$g/ml of ionomycin or 50 $\mu$g/ml of amphotericin B. Cells were permeabilized with saponin prior to staining with rabbit anti-ASP or nonimmune rabbit serum (NRS). The markers are set to exclude from positive analysis 98% of the cells stained with NRS.

### *Correlation of ASP Staining with Cells Displaying DNA Loss (Figure 7)*

Although the ploidy of $B_3B_7$ cells is somewhat unstable (Du Pasquier et al., 1995), those that appear to have either condensed DNA (with associated poor intercalation of PI) or lost DNA (typical of apoptotic cells, see Milner et al., 1995) can be identified from flow cytometry of PI-stained, saponin-treated cells (Figure 7) This figure shows that by 120 hr, ionomycin treatment has induced a significant increase (compared with medium controls) in cells containing "sub-G1" level of DNA. By backgating on PI-stained cells, it can be seen that those with sub-G1 content of DNA are predominantly $ASP^+$ (77% of medium-cultured cells and 90% of ionomycin-treated cells). In contrast, those cells cultured in medium for 120 hr that had normal (G1) content of DNA (located in region A) contain only a very low percentage (~5%) that are $ASP^+$. Interestingly, the relatively few ionomycin-treated cells still in region A now contain a high proportion (69%) that are ASP-positive; these could represent apoptotic cells in G2M or S phase, which have lost DNA and now appear in the G1 region, since this phenomenon has been seen previously with Burkitt lymphoma cells (Milner et al., 1995).

### Western Blotting

Figure 8A illustrates the outcome of serum deprivation over a 336-hr period on 15/0 cells. Apoptosis levels measured by analysis of light scatter reveal a rather high (20%) level of apoptotic 15/0 cells at the time of seeding, and then a gradual increase in apoptotic levels from 120 hr onwards. Western blotting of lysates from these cells at the time of seeding showed no apparent reactivity of anti-ASP, but by 120 hr following seeding, a prominent 66-kD band was recognized. By 336 hr of serum deprivation, the 66-kD band was particularly noticeable and additional bands (including one at 45-kD) were now evident.

Western blots of lysates obtained from $B_3B_7$ cells at various time points during culture in either serum-deprived conditions or following treatment with ionomycin, together with apoptotic levels, are shown in Figure 8B. Consideration of the Western blots allows a number of interesting observations to be made. As with the 15/0 cells, a major band at 66-kD appears in $B_3B_7$ cells induced to apoptose by either serum deprivation or ionomycin treatment. Additional bands are induced by both treatments, with molecular weights of approximately 100, 50, 45, and 40-kD. The induced 45-kD band appears to be only a minor component in these particular apoptotic $B_3B_7$ cells. A 35-kD band is present at low levels even in $B_3B_7$ cells displaying minimal apoptosis, but this band appears to become more intense as apoptotic levels increase. Compared with serum deprivation, ionomycin induces a more rapid emergence of protein bands identified by the ASP antibody. However, by late times of culture, when light-scatter characteristics indicate high numbers of apoptotic cells, similar levels and sizes of ASP proteins are detected in Westerns of both serum-deprived and ionomycin-treated samples, findings that are in agreement with the flow cytometric data shown in Figure 4.

Figure 9 compares Western blots of lysates taken from recently seeded $B_3B_7$ cells (lane 1): those treated for 7 days with ionomycin (to induce extensive apoptosis; (lane 2) and those induced to necrose

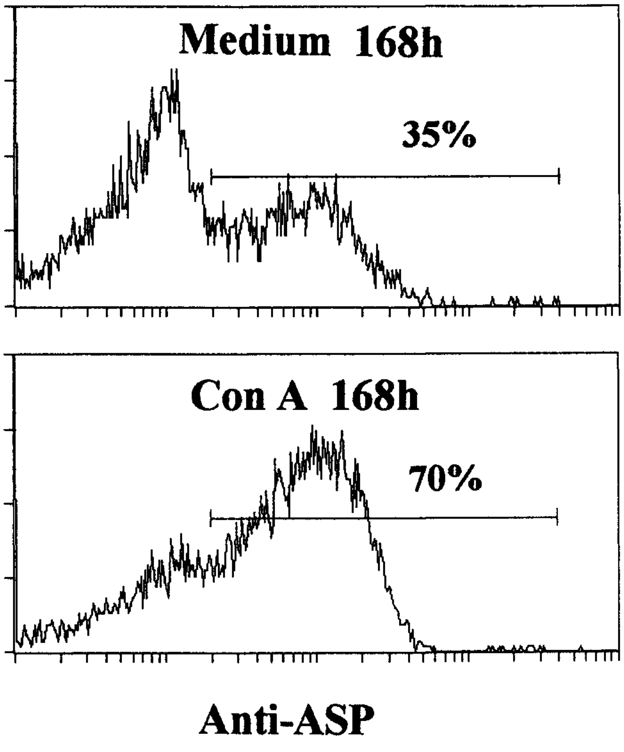

FIGURE 6 Representative flow cytometric traces comparing the percentages of ASP⁺ splenocytes following culture for 168 hr in medium or 1 μg/ml of Con A. Cells were permeabilized prior to staining with anti-ASP. Markers were set as in Figure 5.

following 24-hr treatment in amphotericin (lane 3). (At the time cell lysates were prepared, only 4% of recently seeded lymphocytes were recorded as apoptotic by light-scatter analysis [i.e. in zone D of Figure 1], whereas 52% of the 7-day ionomycin-cultured cells had apoptotic light-scatter profiles. Ninety-six percent of the amphotericin-treated (necrotic) cells displayed reduced forward light scatter, but no increase in side scatter.) The Westerns illustrate that ionomycin induces the appearance of several protein bands detected by the rabbit anti-ASP antibody, but not with nonimmune rabbit serum. In contrast, necrotic $B_3B_7$ cells fail to display any increase in ASP proteins over the level found in the viable cultures. As in previous experiments, major bands at 66 and 45-kD were detected with anti-ASP. Interestingly, the 45-kD band was more highly expressed than the 66-kD one, which mirrors the pattern found in human Burkitt lymphoma cells. It remains to be determined why the relative intensities of the 66- and 45-kD bands in apoptotic $B_3B_7$ cells vary between experiments.

The reliability of ASP for monitoring apoptotic levels in $B_3B_7$ cells was confirmed by use of the TUNEL assay, which identifies fragmented DNA in cell smears. Thus, recently seeded lymphoid tumor cells were only 1% TUNEL$^+$, in contrast to 64% TUNEL$^+$ cells after 7-day ionomycin treatment. Just 9% amphotericin-treated cells were TUNEL$^+$.

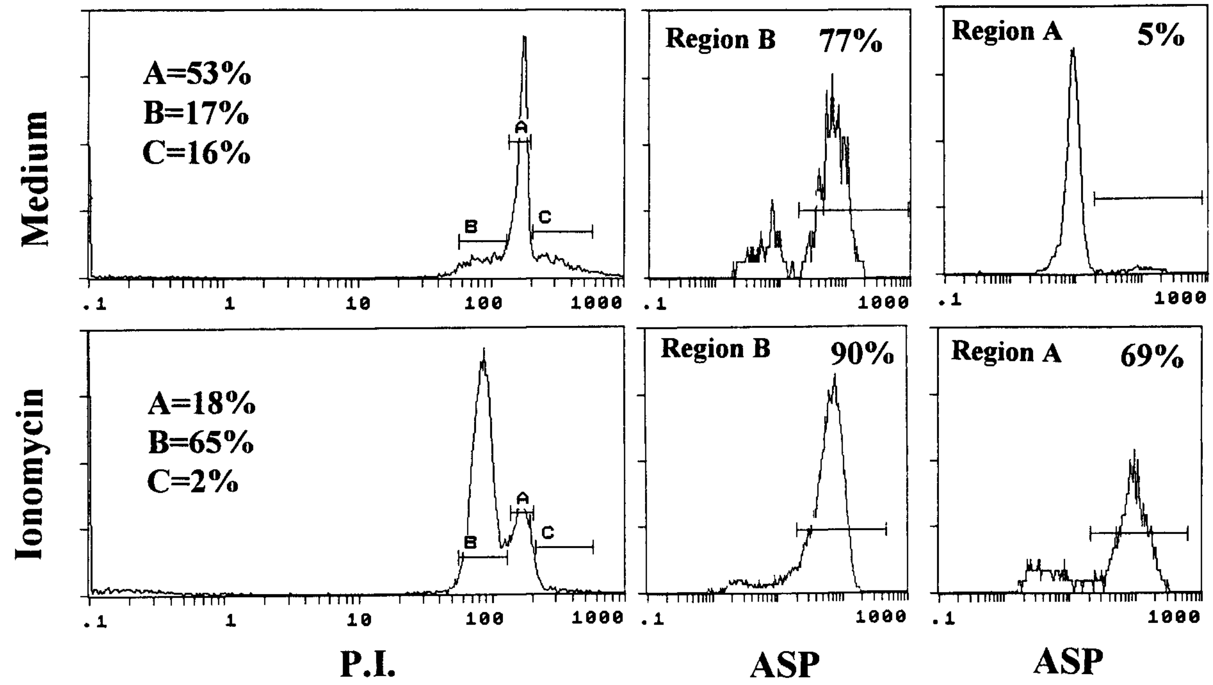

FIGURE 7 Representative flow cytometric analysis revealing association of ASP staining with cells displaying condensation or loss of DNA. $B_3B_7$ cells were taken for FACS analysis 120 hr after seeding to flasks and culture in either medium (top row of histograms) or 10 $\mu$g/ml of ionomycin (bottom row of histograms). Cells were permeabilized with saponin prior to staining for presence of ASP and for DNA content. The histograms at the left show propidium iodide (PI)—staining patterns, which reflect the different content of DNA in cells at various phases of cell cycle: Region A mainly corresponds to cells in the G1 phase, region B to cells that have "sub-G1" DNA content, region C to cells in S phase or G2/M. Ionomycin treatment results in a significantly higher proportion of cells with sub-G1 DNA content, indicative of increased apoptosis. Histograms at the center and right show percentages of ASP$^+$ cells from regions B and A, respectively, of PI traces. For medium-cultured cells, correlation between positive ASP staining (77%) and DNA loss/condensation (cells gated from region B) and the converse lack of ASP staining (5%) in cells from gate A is clearly evident. Although 90% ionomycin-treated cells from sub-G1 region B are ASP$^+$, some 69% from region A are also ASP$^+$.

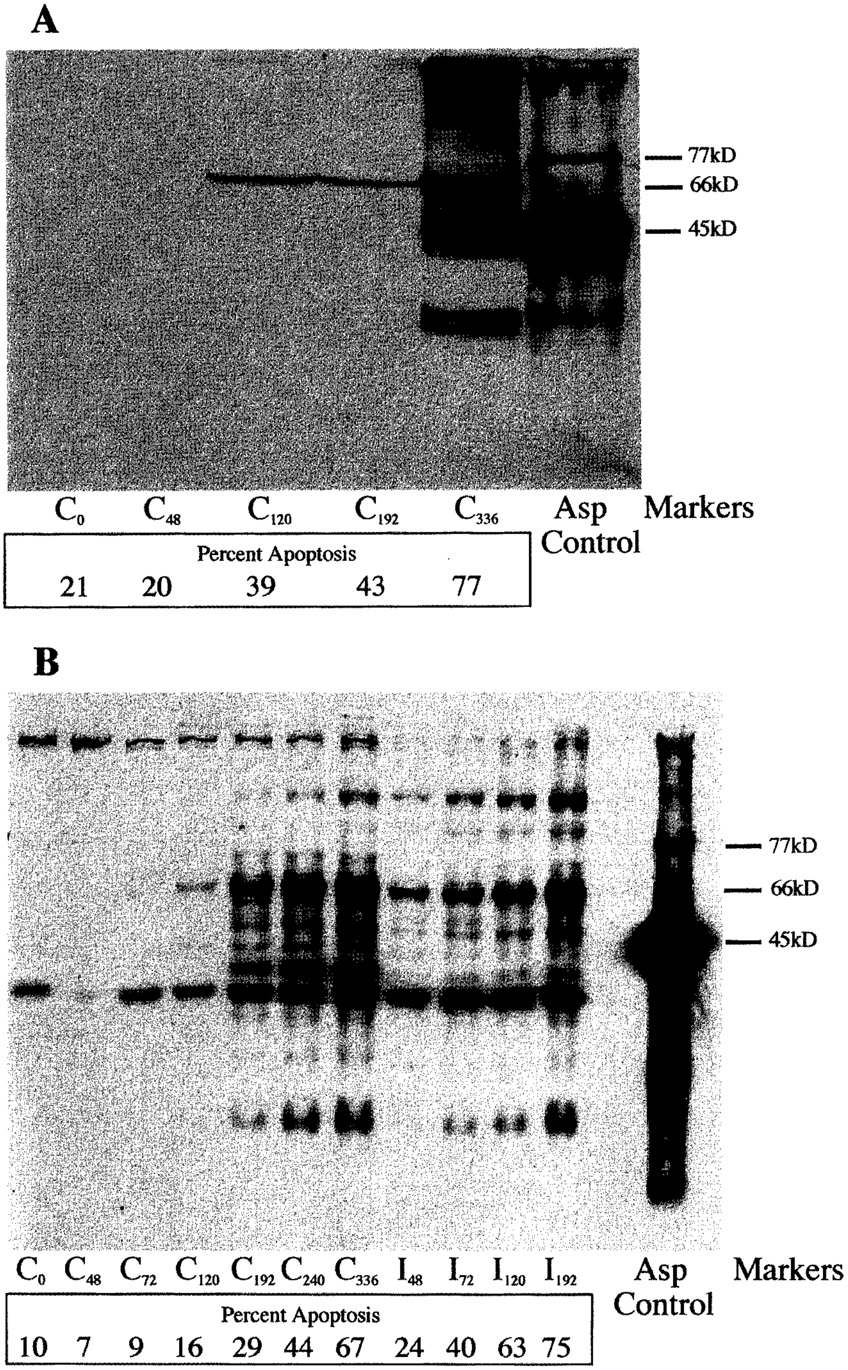

FIGURE 8 Western blots of 15/0 (A) and $B_3B_7$ (B) lymphoid tumor cells showing appearance of protein bands identified by anti-ASP antibody in lysates from cells following either serum deprivation ($C_0$-$C_{336}$ = hours following seeding to culture medium) or ionomycin treatment ($I_{48}$-$I_{192}$ = hours following treatment with 1 $\mu$g/ml ionomycin). Levels of apoptotic cells (measured by light-scatter analysis, as illustrated in Figure 1) in these same cultures are also shown. Burkitt lymphoma cells are the mammalian ASP control in the Westerns, the main ASP band having a molecular weight of 45 kD. The data are representative of duplicate experiments.

### DISCUSSION

DNA loss/ fragmentation has been employed to monitor mitogen-driven apoptosis of lymphocytes in *Xenopus* (Grant et al., 1995), to investigate the increased level of T-lymphocyte apoptosis occurring at metamorphosis (Ruben et al., 1994; Barker et al., 1997) and to study immune-mediated tail loss in this amphibian (Izutsu et al., 1996). The experiments presented here, while revealing that DNA loss can be

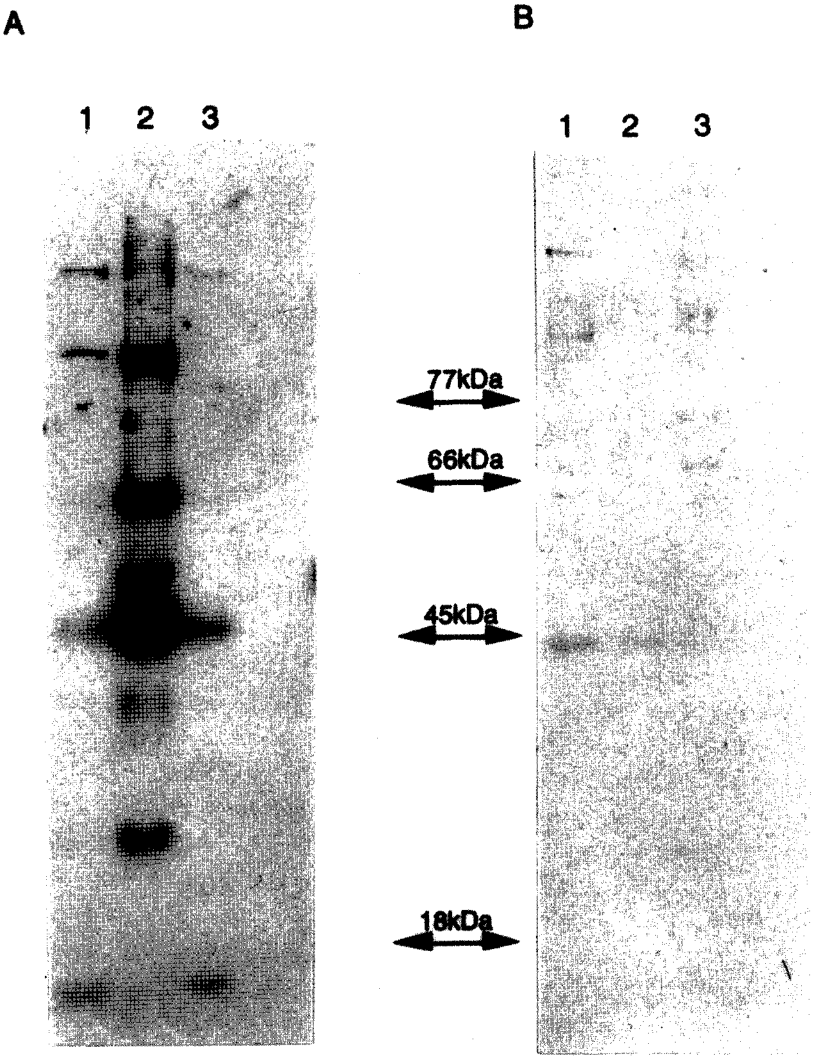

FIGURE 9 Western blots of viable (recently seeded) $B_3B_7$ lymphoid tumor cells (lane 1), in comparison with apoptotic lymphoid tumor cells (168 hr after 1 μg/ml ionomycin treatment. lane 2) and necrotic tumor cells (24 hr after 50 μg/ml amphotericin B. lane 3). *Levels of apoptotic cells (monitored by TUNEL assay) in these same three cultures were 1, 64, and 9%, respectively.* (A) Rabbit anti-ASP was used to identify ASP protein bands in lysates from the three cell populations. (B) The same fractionated proteins were here incubated with control, non-immune rabbit serum; lanes as in 9(A).

used as a marker of ionomycin-induced apoptosis for *Xenopus* tumor cells, introduce a novel assay for monitoring programmed cell death in this amphibian. Specifically, we show that a polyclonal antibody raised against a synthetic peptide of human *c-jun*, known to detect ASP in apoptosing mammalian cells (Grand et al., 1995), is able to identify *Xenopus* lymphoid tumor cells and nontransformed splenocytes undergoing apoptosis. ASP expression in *Xenopus* lymphoid cells and splenocytes can be induced by serum deprivation, but more effectively by incubation with ionomycin; Con A can also induce ASP expression in splenocytes. Cells induced to necrose by the membrane-disrupting fungicide, amphotericin B, failed to become $ASP^{+}$. This finding emphasizes that apoptosis and necrosis are completely separate death pathways in *Xenopus*, as in humans.

For immunostaining of ASP to constitute a valuable and rapid method for detecting apoptosis in *Xenopus*, it should be applicable not only to fluorescence microscopic analysis of cell smears, but also to flow cytometric analysis of cell suspensions. Although this would appear to be the case, the reasons why a significantly higher proportion of lymphoid tumor cells were identified as ASP-positive by flow cytometry than by fluorescence microscopy needs to be resolved. Undoubtedly, the cytometer is more sensitive at fluorescence detection than microscopy and therefore possibly our cytometric analyses detect lower intensities of ASP expression, such as might be found in early apoptotic cells, which have yet to show pycnotic nuclei. On the other hand, the "ASP" signal seen in flow cytometry may be due to immunodetection both of genuine ASP and also of *c-jun*, against which the anti-ASP antibody was raised. The *c-jun* is localized in the nucleus and it is known that it can be upregulated at the onset of apoptosis (Collotta et al., 1992). However, we should point out that no nuclear staining was detected in fluorescence microscopic examination of cytospins of apoptotic $B_3B_7$ cells.

It seems likely, in view of its cytoplasmic distribution, and correlation both with cells displaying pycnotic nuclei and those with reduced DNA content, that a major epitope in *Xenopus* cells recognized by the ASP antibody is in fact ASP, which has been sufficiently conserved between mammals and amphibians. Our finding that the ASP antibody recognizes several protein bands in Western blots of apoptosing $B_3B_7$ and 15/0 *Xenopus* lymphoid tumor cells is similar to the situation in apoptosing mammalian cells, where in addition to the major ASP protein of 45-kD molecular weight, several other components of between 20 and 150-kD have been observed, suggesting a family of apoptosis-specific proteins (Grand et al., 1995). Recently, cDNA encoding the 32-kD component of this protein family has been cloned from a human expression library and this has significant homology to a yeast gene essential for autophagy (Hammond et al., 1998). As yet, it is unknown whether this gene encodes all ASP species. It is conceivable that the variety of ASP proteins seen in Westerns arise through posttranslational modification of the 32-kD ASP. Western blots of apoptosing *Xenopus* tumor cells, while indicating emergence of a protein of 45-kD, reveal this is not always the major induced band recognized by the ASP antibody. Molecular studies are now required to determine the nature of the various bands seen in apoptosing *Xenopus* lymphoid tumor cells.

In summary, we have been able to detect emergence of protein(s) in apoptotic but not necrotic *Xenopus* thymus tumor cell lines and also in apoptotic splenocytes, by use of an anti-*c-jun* polyclonal antibody, which has been previously employed to identify ASP in apoptosing mammalian cells. Detection of apoptotic proteins in *Xenopus* has been possible not only by fluorescence microscopy of fixed cell smears and by Western blot analysis of cell lysates, but also by flow cytometric analysis of permeabilized unfixed cell suspensions, the latter in itself being a novel finding (contrast methodologies used by Grand et al., 1995). This new and simple methodology for identifying apoptotic *Xenopus* cells should expedite studies on the development of the *Xenopus* immune system, such as those investigating the extensive restructuring of immune system cells at metamorphosis (Flajnik et al., 1987; Rollins-Smith and Cohen, 1996) and others (Horton et al., 1996b,

1997a, 1997b( studying the mechanism of tumor-cell cytotoxicity induced by cytolytic lymphocytes.

### MATERIALS AND METHODS

### Tumor-Cell Maintenance and Induction of Apoptosis or Necrosis

The tumor-cell lines $B_3B_7$ and 15/0 were a generous gift from Dr. Louis Du Pasquier. $B_3B_7$ cells are derived from a thymus tumor found in partially inbred, MHC homozygous *Xenopus laevis* family *ff* (Robert et al., 1994), whereas the 15/0 cell line is derived from a thymus tumor from an LG15 (*X. laevis/X. gilli* clone 15) clonal *Xenopus*. In our laboratory, these lymphoid tumor-cell lines are maintained in the *Xenopus* tumor-cell medium at 27°C, subculturing when cell numbers approach $2.0 \times 10^6$/ml to maintain a high proportion of viable cells, new flasks being seeded at approx $2 \times 10^5$/ml. The tumor-cell medium has the following composition: 400 ml serum-free medium (as given below) diluted to amphibian strength with 120 ml triple-distilled water and supplemented with 40 ml supernatant from the A6 amphibian kidney-cell line (Rafferty, 1969), 10 ml decomplemented fetal-calf serum (FCS from Advanced Protein Products), and 200 $\mu$g/ml kanamycin (Gibco). The 15/0 cells were additionally supplemented with 0.25% *Xenopus* serum. Serum-free medium comprises 500 ml Iscove's medium, 5 ml 100× nonessential amino acids, 50 $\mu$g/ml penicillin/streptomycin (all from Gibco), 5 $\mu$g/ml insulin (Sigma), 50 mmol mercaptoethanol (Sigma), and 1.5% Primatone (Roche).

$B_3B_7$ or 15/0 cells were seeded to culture flasks at $2\text{-}3 \times 10^6$ cells in 10 ml tumor medium, and then incubated at 18-20°C since our preliminary observations revealed that cells cultured at this lower temperature were more robust (than those cultured at 27°C) for the purpose of cytospin preparation. *Apoptosis* was induced either by serum deprivation, where cells were kept for up to 14 days without subculture, or by treatment with either 1 or 10 $\mu$g/ml ionomycin (Sigma). *Necrosis* was induced by addition of 50 $\mu$g/ml amphotericin B ("Fungizone" from Gibco). (Additional experiments to the ones reported here on apoptosis induction in $B_3B_7$ cells were also carried out at 27°C; although kinetics of events were more rapid, these studies yielded comparable data.)

### Splenocyte Apoptosis/Necrosis

Splenocyte suspensions ($2 \times 10^6$/ml) from outbred *X. laevis* were depleted of erythrocytes by Ficoll density-gradient centrifugation. One milliliter aliquots in tumor medium (see earlier) were added to 24-well plates and cultured at 27°C. Apoptosis was induced by 72-hr treatment with 1.0 $\mu$g/ml ionomycin, and by 7-day culture in either medium alone (serum deprivation) or in the presence of the T-cell mitogen concanavalin A (1.0 $\mu$g/ml). Necrosis was induced by 3-day treatment with 50 $\mu$g/ml amphotericin B.

### Anti-ASP Antibodies

Two anti-ASP antibodies have been used in our experiments with comparable results. Both are rabbit polyclonal antibodies raised against synthetic peptides of *c-jun* proteins. Initial studies were with Ab-2 (anti-*c-jun*/AP-1) antibody (Oncogene Research Products), raised against a synthetic peptide corresponding to residues 73-87 of human *c-jun*. Since recent batches of this antibody have failed to stain both human and *Xenopus* ASP, we are therefore currently using the SC-45 (*c-jun*/AP-1[N]) antibody (Santa Cruz Biotechnology), raised against a similar peptide of mouse *c-jun*. This reproducibly stains human and *Xenopus* ASP. (A number of other commercially available antibodies to *c-jun* have been tested that fail to cross-react with mammalian ASP [Grand et al., 1995]).

### Fluorescence Microscopy of Tumor Cells

*ASP Staining.*

Tumor cells were transferred to microscope slides by use of a Cytospin (Shandon). $1 \times 10^5$ cells in 100 $\mu$l 40%-FCS-supplemented amphibian PBS (APBS) were loaded into cuvettes and transferred to APBS (40%-FCS)-prewetted slides by centrifugation at 600

rpm for 5 min. After air drying, slides were fixed in acetone for 10 min, then stored at −80°C until needed. Slides were transferred to a moist chamber, blocked with APBS + 1% BSA, then stained for 45 min with 50 $\mu$l anti-ASP antibody (1:100). Normal rabbit serum provided a negative staining control (not shown). After washing in 0.1% BSA-supplemented APBS, primary antibody binding was visualized by staining for 30 min with 50 $\mu$l FITC-conjugated polyclonal goat anti-rabbit-Ig antibody (1:160) (Sigma), adsorbed prior to use with 1:20 dilution of *Xenopus* serum. Slides were again washed well and then counterstained for 2 min with 40 $\mu$g/ml propidium iodide (PI) in APBS. Washed slides were finally mounted with coverslips using a glycerol-based mountant.

*Acridine-Orange Staining.*

Acridine orange (Sigma) at 10 $\mu$g/ml in APBS was added to a pellet of 2 $\times$ $10^5$ APBS (0.1% BSA)-washed tumor cells. After gentle resuspension, 10 $\mu$l cell suspension was pipetted onto a slide and the sample immediately examined by fluorescence microscopy.

*Microscopy.*

Coded slides were examined with a Nikon Optiphot fluorescence microscope with incident light illumination, using a FITC-filter set. At least 500 cells in minimally 10 fields were counted on duplicate slides, to obtain large enough sample sizes. The number of $ASP^+$ cells or cells with pycnotic nuclei (latter identified by staining with PI or acridine orange) counted in each field was expressed as a proportion of the total number of cells in that field, and the mean proportion in all the fields was calculated.

### Flow Cytometry

$B_3B_7$ lymphoid-cell lines or splenocytes were washed twice in FACS medium (APBS, containing 0.1% BSA and 0.1% $NaN_3$). Some cells were then permeabilized by 30-sec treatment of 1-2 $\times$ $10^6$ pelleted cells with 100 $\mu$l 0.1% saponin (Sigma) in FACS medium, prior to addition of excess FACS medium. Following two washes, the permeabilized cells and others left untreated were aliquoted into a 96-well plate (2 $\times$ $10^5$ cells/well), then incubated with either 1:100 rabbit anti-ASP (see earlier) or with 1:100 normal rabbit serum (NRS) to provide control staining levels. Following another two washes, cells were stained in 1:160 FITC-conjugated goat anti-rabbit secondary antibody (see earlier) and again washed. Immediately prior to analysis in the flow cytometer, antibody-stained cells (1 $\times$ $10^6$/ml) were counterstained with PI (40 $\mu$g/ml). Markers were set to exclude 98% of cells stained with normal rabbit serum from positive analysis of ASP staining levels. Ten thousand tumor cells per sample were analyzed on a Coulter XL flow cytometer.

*Nonpermeabilized Cells.*

*Nonpermeabilized* cells were used both for assessing apoptosis as judged by alteration in forward/side-scatter properties (see Milner et al., 1995) and also for monitoring the proportion of cells with "naturally occurring" leaky plasma membranes, as revealed by propidium iodide (PI) entry.

*Permeabilization.*

*Permeabilization* allows intracellular staining by anti-ASP of all $ASP^+$ cells, including many that before saponin treatment would not have stained because they had intact plasma membranes. Permeabilization also enables PI to monitor total DNA and hence identify cells in various phases of cell cycle, including apoptotic cells, many of which should display reduced DNA levels.

### Western Blotting of Tumor-Cell Lysates

Cells were harvested, washed once in PBS, pelleted by centrifugation, and the pellets stored at −20°C. Thawed pellets were lysed in 9 M urea, 50 mM Tris-HCL, pH 7.4, 0.15 M $\beta$-mercaptoethanol, then sonicated. Aliquots containing equal amounts of protein (50 μg) were subjected to 12% SDS-PAGE. The fractionated proteins were transferred to nitrocellulose membranes that were incubated with either nonimmune rabbit serum (diluted 1:200) as control reagent, or rabbit anti-ASP antibody diluted 1:200, and then with anti-rabbit horseradish peroxidase-conjugated antibody (Dako). Target proteins were visualized using ECL reagent (Amersham International)

### TUNEL Assay on Tumor Cells

Samples of $B_3B_7$ cells to be monitored for ASP proteins by Western blotting were transferred to slides and assessed for apoptosis using the TUNEL (*T*dT-mediated d*U*TP *N*ick-*E*nd *L*abeling) assay, to end-label fragmented DNA found in apoptotic cells. A Boehringer "*in situ* cell death detection kit" was used in this assay. The manufacturer's instructions were followed exactly, except that samples were incubated for just 15 min at 37°C.

### *Acknowledgments*

Research grants from the Leverhulme Trust and the University of Durham (to JDH) are gratefully acknowledged. AM, EH, and RG are funded by the Cancer Research Campaign. We are extremely grateful to Dr. Louis Du Pasquier, Basel Institute for Immunology, for providing the *Xenopus* lymphoid tumor cells. Thanks also go to Paul Sidney, David Hutchinson, and Ralph Minter for photographic assistance.

### *References*

An S., and Knox K.A. (1996). Ligation of CD40 rescues Ramos-Burkitt lymphoma B cells from calcium ionophore- and antigen receptor-triggered apoptosis by inhibiting activation of the cysteine protease CPP32/Yama and cleavage of its substrate PARP. *FEBS Lett.* **386**, 115-122.

Barker K.S., Davis A.T., Li B., and Rollins-Smith L.A. (1997). *In vitro* studies of spontaneous and corticosteroid-induced apoptosis of lymphocyte populations from metamorphosing frogs/RU486 inhibition. *Brain, Behav. Immun.*, in press.

Berke G. (1995). Unlocking the secrets of CTL and NK cells. *Immunol. Today* **16**, 343-346.

Colotta F., Polentarutti N., Sironi M., and Mantovani A. (1992). Expression and involvement of *c-fos* and *c-jun* protooncogenes in programmed cell death induced by growth factor deprivation in lymphoid cell lines. *J. Biol. Chem.* **267**, 18278-18283.

Duke R.C. (1996). Cell suicide in health and disease. *Sci. Am.* **275**, 48-55.

Du Pasquier L., and Robert J. (1992). *In vitro* growth of thymic tumor cell lines from Xenopus. *Dev. Immunol.* **2**, 295-307.

Du Pasquier L., Wilson M., and Robert J. (1996). The immune system of *Xenopus*: Special focus on B cell development and immunoglobulin genes. In Biology of *Xenopus*, Tinsley R.C., and Kobel H.R. Eds. (Oxford: Oxford University Press), pp. 301-313.

Flajnik M.F., Hsu E., Kaufman J.L., and Du Pasquier L. (1987). Changes in the immune system during metamorphosis of Xenopus. *Immunol. Today* **8**, 58-64.

Grand R.J.A., Milner A.E., Mustoe T., Johnson G.D., Owen D., Grant M.L., and Gregory C.D. (1995). A novel protein expressed in mammalian cells undergoing apoptosis. *Exp. Cell. Res.* **218**, 439-451.

Grant P., Clothier R.H., Johnson R.O., Schott S., and Ruben L.N. (1995). The time course, localization and quantitation of T- and B-cell mitogen-driven apoptosis in vivo. *Immunol. Lett.* **47**, 227-231.

Gregory C.D. (1995). Apoptosis and the Immune Response (New York: John Wiley).

Hammond E.M., Johnson G.D., Brunet C.L., Parkhill J., Milner A.E., Brady G., Gregory C.D., and Grand R.J.A. (1998). Homology between a human apoptosis specific protein and the product of APG5, a gene involved in autophagy in yeast. *FEBS Lett.* **425**, 391–395

Horton J.D., Horton T.L., and Ritchie P. (1996a). Immune system of Xenopus: T cell biology. In The Biology of *Xenopus*, Tinsley, R.C., and Kobel, H.R., Eds. (Oxford: Oxford University Press), pp. 279-299.

Horton T.L., Gravenor I., Ritchie P., Minter R., Watson M.D., and Horton J.D. (1997b). Use of thymectomized *Xenopus* to study extra-thymic T cell and NK cell evolution. *Dev. Comp. Immunol.* **21**, 237.

Horton T.L., Ritchie P., Watson M.D., and Horton J.D. (1996b). NK-like activity against allogeneic tumour cells demonstrated in the spleen of control and thymectomized Xenopus. *Immunol. Cell Biol.* **74**, 365-373.

Horton T.L., Ritchie P., Watson M.D., and Horton J.D. (1997a). NK cell evolution: Studies on Xenopus. *Biochem. Soc. Trans.* **25**, 263S.

Izutsu Y., Yoshizato K., and Tochinai S. (1996). Adult-type splenocytes of *Xenopus* induce apoptosis of histocompatible larval tail cells *in vitro*. *Differentiation* **60**, 277-286.

Kerr J.F.R., Wyllie A.H., and Currie A.R. (1972). Apoptosis: A basic biological phenomenon with wide-ranging implications in tissue kinetics. Brit. J. Cancer **26**, 239-257.

Martins L.M., and Earnshaw W.C. (1997). Apoptosis: Alive and kicking in 1997. *Trends Cell Biol.* **7**, 111-114.

Milner A.E., Wang H., and Gregory C.D. (1995). Analysis of apoptosis by flow cytometry. In Applications of Flow Cytometry in Biotechnology, Al-Rubai M., Ed. (New York: Marcel Dekker), pp. 193-209.

Nagata S., (1997). Apoptosis by death factor. *Cell* **88**, 355-365.

Osborne B.A. (1996). Apoptosis and the maintenance of homeostasis in the immune system. *Curr. Opinion. Immunol.* **8**, 245-254.

Rafferty K.A. (1969). Mass culture of amphibian cells: Methods and observations concerning stability of cell type. In Biology of Amphibian Tumors, Mizell M., Ed. (New York: Springer-Verlag), pp. 52-81.

Robert J., Guiet C., and DuPasquier L. (1994). Lymphoid tumors of *Xenopus laevis* with different capacities for growth in larvae and adults. *Dev Immunol.* **3**, 297-307.

Rollins-Smith L.A., and Cohen N. (1996). Metamorphosis: An immunologically unique period in the life of the frog. In Metamorphosis: Postembryonic Reprogramming of Gene Expression in Amphibian and Insect Cells., Gilbet L.I., Atkinson B.G., and Tata J., Eds. (New York:Academic Press), pp. 625-646.

Ruben L.N., Ahmadi P., Johnson R.O., Bucholz D.R., Clothier R.H., and Shiigi S. (1994). Apoptosis in the thymus of developing Xenopus laevis. *Dev. Comp. Immunol.* **18**, 343-352.

